# Why Do Some Primate Malarias Relapse?

**DOI:** 10.1016/j.pt.2016.08.014

**Published:** 2016-12

**Authors:** Nicholas J. White

**Affiliations:** 1 Mahidol Oxford Tropical Medicine Research Unit, Faculty of Tropical Medicine, Mahidol University, Bangkok 10400, Thailand; 2 Centre for Tropical Medicine and Global Health, Nuffield Department of Clinical Medicine, Churchill Hospital, Oxford, OX3 7LJ, United Kingdom

## Abstract

Relapse may have evolved in malaria as a mechanism to avoid suppression by more virulent species in mixed infections, thereby increasing transmission opportunities. Later evolution of long latency in *Plasmodium vivax* was a necessary adaptation as early hominins moved to colder areas with shorter mosquito breeding seasons. Genetic diversity was maintained through heterologous hypnozoite activation.

## Sleeping Parasites

Sporozoan (apicomplexan) parasites have developed many different mechanisms to facilitate persistence in their hosts. Plasmodial parasites persist through continuous multiplication in blood for extended periods. In several of the primate malarias an alternative strategy has evolved. Some of the sporozoites persist as sleeping forms or ‘hypnozoites’, which lie dormant in the liver hepatocytes for long periods after inoculation and wake weeks or months after inoculation [1]. These relapses have a remarkable periodicity [2]. In humans, *Plasmodium vivax* is the main cause of relapsing malaria. There are an estimated 100–400 million cases each year mainly in Asia, Oceania, the horn of Africa and South America. *P. vivax* is much more difficult than *Plasmodium falciparum* to eliminate, largely because of relapses which are a major contributor to morbidity. But why do *P. vivax, Plasmodium ovale* and some other primate malarias relapse? What evolutionary pressure led to this remarkable adaptation? I propose that it arose in these primate parasites as an adaptive ‘defensive’ response to concomitant symptomatic infections with virulent parasites such as *Plasmodium falciparum* or other now extinct species.

## Epidemiology and Early Evolution

Premunition to *P. vivax* develops more rapidly than to *P. falciparum* [3]*.* This is largely because of frequent relapse–a single inoculation giving rise to multiple episodes of illness in the case of *P. vivax* but only one in the case of *P. falciparum.* Before the era of modern medicine, this difference would presumably have been smaller, as primary infections of either species would have persisted in the blood for weeks or months, unless challenged by superinfection. In all epidemiological contexts, relapse is an important contributor to *P. vivax* incidence and prevalence. In tropical regions, relapse intervals are short and frequent. The first relapse parasites begin to emerge from the liver approximately 4 weeks after sporozoite inoculation (2 weeks after the primary illness) and reach patency (detectable parasitaemias) approximately 1 week later [2]. The infection becomes transmissible just before this. In temperate regions, where mosquito breeding seasons are short and *P. vivax* is obliged to overwinter in humans, the intervals to first relapse are much longer [2]. Adaptation to temperate climes would have occurred later as hominins ventured further north from the tropical areas long after the evolution of the relapse mechanism.

If *P. vivax* emerged as a primate malaria parasite in the forests of Africa, then it would always have had to contend with more virulent, more rapidly developing, and generally dominant malaria parasites with which it shared mosquito vectors and primate hosts. When *P. falciparum* and *P. vivax* are inoculated together, then *P. falciparum* usually dominates 4, 5; it develops more rapidly in the liver and then the symptomatic blood stage infection suppresses asexual multiplication of *P. vivax.* Despite this, there is a remarkably high rate of mixed infections with the two species, so both are commonly transmitted together 6, 7. In higher transmission settings there is cross-species regulation of parasite densities [7]. Through a range of different epidemiological circumstances, the two malaria parasites are effectively in competition. Under similar conditions of competition the relapse mechanism evolved in the predecessors of today's primate plasmodial parasites.

## Intrahost Competition

In contrast with *P. falciparum, P. vivax* gametocytogenesis occurs immediately, so the infection is transmissible as soon as it reaches densities in blood that are sufficient for gametocyte ingestion by feeding anopheline mosquitos. But as sexual parasites are derived from asexual parasites, anything that suppresses asexual multiplication reduces overall transmissibility of the infection. Transmissibility is greatest during higher-density infections which generate larger numbers of gametocytes. Competition with the predecessor of *P. falciparum* (or other more virulent parasites, such as the parasite which drove Duffy negativity to fixation in much of Africa) in human primate ancestors provided an evolutionary selection pressure on other malaria parasites to avoid suppression in the acute phase of mixed malaria infections.

During the acute phase of the infection, before the acquisition of disease controlling immunity, nonspecific host defence mechanisms comprising fever, proinflammatory cytokine release, and splenic activation contain the infection in most cases. As a byproduct this limits the expansion of any concomitant infection. Although *P. vivax* is a more potent inducer of this host-defence response than *P. falciparum*, we know from the simultaneous inoculation experiments in human volunteers and malaria therapy that *P. falciparum* usually predominates 4, 5, 6*.* Thus *P. vivax* gets hit in the crossfire and retreats. But once the acute illness has subsided there is less impediment to *P. vivax* multiplication. In many tropical areas, the first relapse coincides with a decline in *P. falciparum* parasitaemia in untreated infections, as antibodies to the predominant *P. falciparum* PfEMP1 variant expressed at the surface of the red blood cell rise [8]. *P. vivax* also has gene families suggesting antigenic variation, and can also persist in the blood for protracted periods in nonimmune individuals, although how it does so is not well understood. However, it seems likely that persistence of a blood stage infection of *P. vivax* at the densities necessary for transmission would result in a significant immune response and, without antigenic variation, clearance of the infection. Disappearance from the blood without providing an ‘immunizing dose’ of infection, under suppression from *P. falciparum* (or its evolutionary precursor) and reappearance weeks or months later is a much more efficient strategy to generate transmissible densities of sexual parasites.

In a mixed infection, the acute illness associated with falciparum malaria suppresses the concomitant blood stages of *P. vivax* reducing their densities and thus transmissibility. The illness also delays or prevents development of any developing *P. vivax* liver stage parasites by reducing the availability of iron through increases in hepcidin (the main hormone regulating iron availability) [9]. As sporozoite inocula are generally thought to be small (median six to ten sporozoites), these two factors reduce the transmission potential of sporozoites that activate immediately after arrival to the liver. Delaying activation until the nonspecific host responses have attenuated or abated would increase the probability of generating and maintaining transmissible parasite densities. This is ‘place betting’. Through this mechanism, *P. vivax* can establish and transmit if there is no coinfection, or retreat and transmit later if there is. It also increases the probability that minor populations in mixed genotype *P. vivax* infections can transmit. Even if a heterologous genotype is outcompeted in the primary infection, while it has hypnozoites in the liver, it still has a chance to transmit every time it relapses. With multiple rolls of the dice, the probability of successful transmission increases. Relapse therefore makes most efficient use of the inoculated sporozoites to optimize the probability of transmission.

## Evolutionary Advantages of Relapse

Systemic febrile illness (such as falciparum or vivax malaria) may activate relapses 2, 10. This links infections of different species and provides a highly effective mechanism for ensuring genetic recombination between unrelated *P. vivax* parasites in times or places where transmission is low. Nonspecific activation by febrile illness or another stimulus may result in the simultaneous development of hypnozoites from the same (homologous) and earlier (heterologous) inoculations. In anopheline vectors feeding during the ensuing blood stage infection, male and female gametes derived from the different inoculations can mate (heterologous recombination) creating genetic diversity 2, 11. This maximizes the opportunities for both transmission (even if there is homologous strain immunity) and immune evasion, and it explains how *P. vivax* maintains high genetic diversity even in areas of very low seasonal transmission. The evolutionary advantage of *P. vivax* linking to *P. falciparum*, whilst avoiding it during the acute phase, may be to exploit transmission conditions optimally. A symptomatic *P. falciparum* infection is unequivocal evidence of the recent availability of vector mosquitoes. For a sleeping hypnozoite, the optimal time to wake is when vector mosquitos are abundant but the fire of *P. falciparum* acute illness has subsided, so that effective *P. vivax* multiplication is unhindered. If the illness associated with relapse is sufficiently severe, this will activate further hypnozoites creating regular periodicity (every 3 weeks approximately). Illness is a sign of inadequate immunity, and that translates into an increased probability that the subsequent relapse can reach patency. When immunity to *P. falciparum* is acquired more slowly than to *P. vivax*, as in many low-transmission settings, then falciparum malaria in adults may wake *P. vivax* hypnozoites that would not be woken by asymptomatic vivax malaria.

As early man moved north to areas where winter temperatures were inhospitable to mosquitos and fell below those allowing sporogony, there was less competition from *P. falciparum*, but there were also fewer opportunities for relapse activation in the short summer transmission season. Early relapse became a wasted transmission opportunity, and a clock evolved so that the relapse came to coincide with next year's anopheline vector abundance. Notably, second relapses were at 3-week intervals as in the tropical strains. Eventually, further north, with even shorter transmission seasons, the opportunity to transmit from the primary illness diminished, and nearly all the inoculated sporozoites became dormant hypnozoites 2, 12. Long-latency *P. vivax* had become long-incubation-period *P. vivax* (*hibernans*).

## References

[1] Garnham P.C.C. (1967). Relapses and latency in malaria. Protozoology.

[2] White N.J. (2011). Determinants of relapse periodicity in *Plasmodium vivax* malaria. Malar. J..

[3] Luxemburger C. (1996). The epidemiology of malaria in a Karen population on the western border of Thailand. Trans. R. Soc. Trop. Med. Hyg..

[4] Boyd M.F., Kitchen S.F. (1937). Simultaneous inoculation with *Plasmodium vivax* and *P. falciparum*. Am. J. Trop. Med..

[5] Mayne B., Young M.D. (1938). Antagonism between species of malaria parasites in induced mixed infections. Public Health Rep..

[6] Mayxay M. (2004). Mixed-species malaria infections in humans. Trends Parasitol..

[7] Bruce M.C., Day K.P. (2003). Cross-species regulation of *Plasmodium* parasitemia in semi-immune children from Papua New Guinea. Trends Parasitol..

[8] Recker M. (2011). Antigenic variation in *Plasmodium falciparum* malaria involves a highly structured switching pattern. PLoS Pathog..

[9] Portugal S. (2011). Host-mediated regulation of superinfection in malaria. Nat. Med..

[10] Shanks G.D., White N.J. (2013). The activation of vivax malaria hypnozoites by infectious diseases. Lancet Infect. Dis..

[11] Noviyanti R. (2015). Contrasting transmission dynamics of co-endemic *Plasmodium vivax* and *P. falciparum*: implications for malaria control and elimination. PLoS Negl. Trop. Dis..

[12] Battle K.E. (2014). Geographical variation in *Plasmodium vivax* relapse. Malar. J..

